# The importance of collecting and sharing scientific publications in pediatric orthopedic surgery from China

**DOI:** 10.1177/18632521251380440

**Published:** 2025-09-18

**Authors:** Federico Canavese, Fritz Hefti, Shlomo Wientroub

**Affiliations:** 1 Orthopedic and Traumatology Department, IRCCS Istituto Giannina Gaslini, Genova, Italy; 2 Dipartimento di Scienze Chirurgiche e Diagnostiche Integrate (DISC), University of Genova, Genova, Italy; 3 Children’s University Hospital of Basel (UKBB), Basel, Switzerland; 4 Orthopedic Surgery, Tel Aviv University, Tel Aviv, Israel

**Keywords:** China, pediatric orthopedics, treatment, outcome, special collection

## Abstract

The *Journal of Children’s Orthopedics* has compiled a special collection of scientific publications from Chinese centers accepted for publication in the journal. Through this collection, the *Journal of Children’s Orthopedics* demonstrates its commitment to promoting global knowledge sharing and collaboration in pediatric orthopedic surgery. The articles in the collection undergo the same rigorous peer review process as other articles. Once a publication is assigned to an issue, it is automatically added to the Special Chinese Collection on the *Journal of Children’s Orthopedics* website, where it can be easily downloaded. The Special Chinese Collection’s open access policy increases the visibility and global reach of *Journal of Children’s Orthopedics* articles, promoting accelerated citations and collaborations. The *Journal of Children’s Orthopedics* is an ideal platform for collecting and disseminating high-quality, relevant scientific publications in pediatric orthopedic surgery from China. The Special Chinese Collection showcases innovative research, encourages knowledge sharing, and fosters cultural exchange, promoting the development of a global community of researchers and clinicians dedicated to advancing the field of pediatric orthopedic surgery and improving children’s lives worldwide.

Pediatric orthopedic surgery is a specialized field focusing on the diagnosis, treatment, and management of musculoskeletal disorders in children. As this field evolves rapidly, it is crucial to collect and disseminate scientific knowledge and research findings from around the world, including China. China is one of the most populous and youngest countries in the world, with only one-third of its 1.4 billion people being over 65 years of age, about 25% being under 14 years of age, and ~10 million children being born each year, resulting in an annual birth rate of about 7/1000 people.^1,2^ Providing bone and joint healthcare to this population is of great significance and depends on professional training and practice.^2,3^

China has made significant progress in improving its healthcare system, particularly in preventive care and early intervention, given its relatively young population. However, pediatric orthopedics remains an underdeveloped field in China. Pediatric orthopedic surgery involves a wide range of congenital, traumatic, infectious, and tumor conditions, but, unlike adult orthopedics, it treats patients who are still developing and require unique surgical approaches and postoperative care.

China is facing a severe shortage of pediatric orthopedic surgeons, and many hospitals and medical institutions do not have departments dedicated to this field. Consequently, most children rely on general orthopedic surgeons for care. This means they miss out on the specialized treatment they need, which can lead to negative outcomes, including permanent disability. The disparity in the concentration of specialists between major urban areas and rural areas exacerbates this issue, as rural areas have limited access to patient care and training opportunities.^1–3^

Despite these challenges, a significant number of Chinese researchers and clinicians have contributed to the global body of knowledge in pediatric orthopedic surgery. This is evident from the growing number of scientific publications in this field. There is a theoretical advantage to conducting scientific research in pediatric orthopedics in China because its patient population is much larger than that in Europe and North America. This is important for conducting clinically relevant studies.

In this context, the *Journal of Children’s Orthopedics* (JCO) has created a special collection of scientific publications from Chinese centers that have been accepted for publication in the journal. Once a publication is assigned to an issue of the JCO, it is automatically added to the Special Chinese Collection on the JCO website, where it can easily be downloaded. The Special Chinese Collection is a valuable platform that showcases the latest research and advancements in pediatric orthopedic surgery originating from Chinese centers. Articles included in the Special Chinese Collection undergo the same rigorous peer review process as other articles. For instance, from January 2021 to August 2025, the JCO received over 130 submissions from Chinese centers. Of those, only 31 were accepted and included in the Special Chinese Collection: 30 original articles and one letter to the editor, for an overall acceptance rate of 23.7%. Furthermore, the articles published in JCO and included in the Special Chinese Collection are open access. As such, they have the advantage of greater visibility and dissemination, and they can reach a global audience. This promotes accelerated citations and collaborations between researchers.

JCO is an ideal platform for collecting and disseminating high-quality, relevant scientific publications from China. The journal has maintained a relatively stable impact factor in recent years. Its latest impact factor is 1.6, the highest among pediatric orthopedics journals, while its 5-year impact factor is 1.7. Overall, JCO is ranked 78th out of 139 orthopedic journals (Q3) and 91st out of 191 pediatric journals (Q2).

By dedicating a Special Chinese Collection to Chinese research, JCO demonstrates its commitment to promoting global knowledge sharing and collaboration in pediatric orthopedic surgery. Publishing in a leading international journal, such as JCO, within the pediatric and adult orthopedic surgery community can increase the visibility of Chinese researchers’ work.

The Special Chinese Collection allows researchers and clinicians worldwide to learn from Chinese specialists and stay informed about the latest advancements in pediatric orthopedic surgery at Chinese institutions. This increased visibility could lead to collaborations with researchers from other countries, facilitate cultural exchange and understanding between Chinese and international researchers, and promote a more global approach to pediatric orthopedic disorders and their treatment. Ultimately, these efforts could improve patient care and outcomes for children with musculoskeletal disorders.

Despite the many challenges, there are significant opportunities for growth and collaboration in pediatric orthopedic surgery between China and the international community. This is evident at international meetings, such as EPOS and POSNA, where the number of Chinese scholars presenting future publications is steadily growing. Through collaboration, researchers and clinicians can advance the field and improve outcomes for children worldwide.

The Special Chinese Collection compiles scientific publications on pediatric orthopedic surgery from Chinese centers. This valuable resource showcases innovative Chinese research, promotes knowledge sharing, and facilitates cultural exchange. By collaborating, Chinese and international researchers can advance the field of pediatric orthopedic surgery and improve care and outcomes for children with musculoskeletal disorders. These initiatives foster a global community of researchers and clinicians dedicated to advancing the field and improving the lives of children worldwide.

## References

[1] Sun X Zhao L Zhao D , et al Development of pediatric orthopedic training and practice in China: administrative, socioeconomic, and cultural impacts. J Pediatr Orthop B 2016; 25(3): 283–287. 27003319 10.1097/BPB.0000000000000271

[2] Dougherty PJ Chen C Zhang Y. CORR^®^ curriculum—orthopaedic education: orthopaedic surgery education in China. Clin Orthop Relat Res 2017; 475(1): 35–38. 27844402 10.1007/s11999-016-5162-z PMC5174074

[3] Lyu X Xu H Feng L , et al Origins and development of pediatric orthopedic surgery in China. J Pediatr Orthop B 2020; 29(5): 415–416. 32371651 10.1097/BPB.0000000000000739

